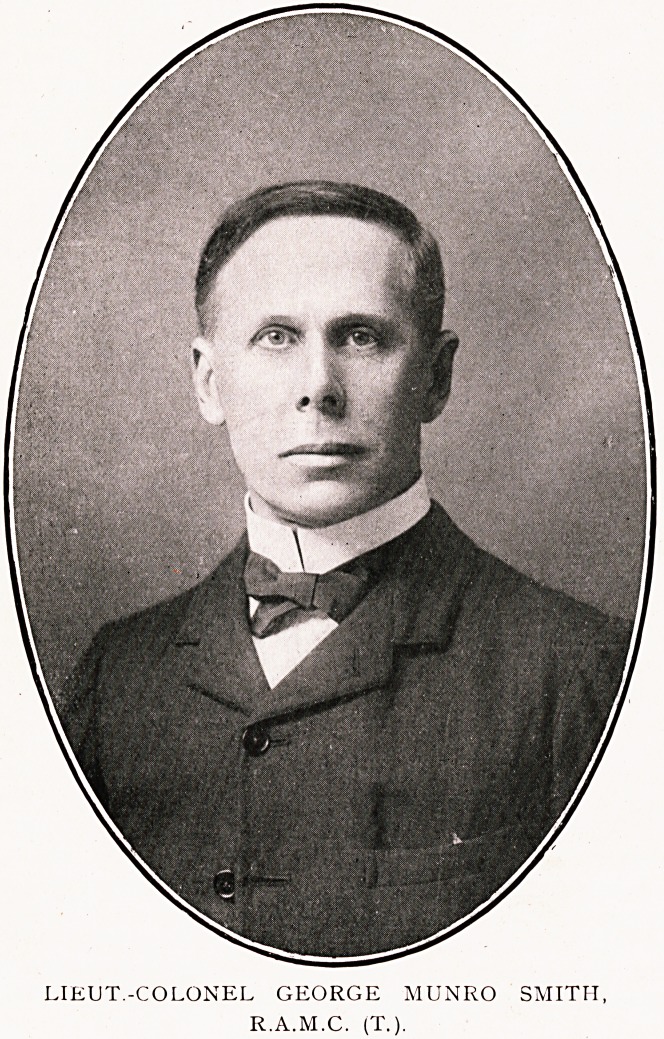# George Munro Smith, M.D. Bris., L.R.C.P., M.R.C.S.

**Published:** 1917-04

**Authors:** 


### GEORGE MUNRO SMITH, M.D. Bris., L.R.C.P., M.R.C.S.

Great was the shock, and much the grief, when it was first made known that Dr. Munro Smith was suffering from an incurable disorder. It was known to himself and some colleagues that for a year or two he had patiently put up with much discomfort from some internal trouble, but when, in a tragic manner whilst operating, he fainted and was taken home ill, it was evident that the strong man was failing, that something serious was going on, and when a few days later we heard that the operator could do no more than relieve by colotomy, it was evident that Munro Smith's days were numbered.

Born in 1856, he died at his house in the Apsley Road on January 13th, 1917, at the comparatively early age of 60, in the prime of life, and with many possible years of useful activity before him. The son of a medical man, he chose his own medical career, and living in Clifton, he naturally was educated at the Clifton College. Much of his future success was doubtless founded on this early training, and his teachers there would have encouraged the indications they found that he was a born naturalist, and a painstaking worker in many other fields of activity.

Afterwards he joined the Bristol Medical School, and there he soon showed his interest in the profession of his choice. He won the Clarke Scholarship, gained the Suple Gold Medal in Medicine and Surgery at the Bristol Royal Infirmary, and was prizeman in various subjects. His medical student's career began in 1873, and afterwards he became medical tutor, and

LIEUT.-COLONEL GEORGE MUNRO SMITH,
R.A.M.C. (T.).

was Demonstrator in Physiology from 1881 to 1887. He then wisely decided to attend a course or courses of Physiology at University College, London, where the work of Sir J. Burdon Sanderson and Drs. Klein and Foster was attracting much attention. There he worked in the laboratory preparatory to his appointment as Lecturer in Physiology to the Bristol Medical School from 1887 to 1893, when he became Professor of Physiology in the University College, Bristol, from 1893 to 1899.

From his early student days he had been connected with the Bristol Royal Infirmary, and from that time onward was always associated with the work of that Institution. From 1889 to 1897 he held the office of Assistant Surgeon, when he became full Surgeon, which office he held for a further term of eleven years, resigning in 1909. During this period he was most faithful in attention to the duties of his offices, and large numbers of patients must have cause to be grateful for his skill and devotion. He was then appointed Consulting Surgeon, which office he continued to hold as long as he lived. He was accordingly connected with the Institution for a period of nearly forty years.

He was held in much esteem by his colleagues and medical friends, and his work was much valued by the students with whom he was so much in contact, for he had long learnt the value of a close and sympathetic relationship between professor and student, and he had the power of winning the affection and confidence of these young men, so greatly aiding in the development of character in the undergraduate. His personality was great, his sympathies were wide and varied, he was a good lecturer, clear in his demonstrations, keen in the service of science, and a naturalist of considerable repute. His devotion to duty was one of his great characteristics which made him popular with his patients and colleagues.

He was President of the Bristol Naturalists' Society in 1910, 1911 and 1912, and wrote many papers for the Society's *Journal*. Of this aspect of his work Mr. C. King Rudge writes as follows: "George Munro Smith was a true lover of nature, a born field naturalist, an accurate observer, and a careful interpreter of facts. He was specially interested in the birds of the Bristol district, more particularly our rarer summer and winter migrants, the nesting of many of which he has recorded in this locality. He was elected a member of the Bristol Naturalists' Society in 1882, and from that time onwards—a period of thirty-five years—he took the keenest interest in the work of the Society, reading many interesting papers at the General Meetings on various subjects. He also frequently exhibited objects of interest, and joined in the discussions at the meetings. Many of his observations and notes on bird life were made on the Downs, the Avon banks, and the flats and marshes by the

Severn shore. Being a skilful photographer, he was often able to illustrate his papers by well-chosen lantern pictures of birds and their nests.

"Munro Smith will be greatly missed at the meetings of the Bristol Naturalists' Society, where his cheery personality, and ready wit were always appreciated by his fellow-members."

His appearance at the meetings of the Medical Dramatic Society was always greatly welcomed, as he was an accomplished actor, especially in parts which gave scope for the manifestation of his natural humorous talents.

He wrote many papers, several published in the *Journal* of the Bristol Medico-Chirurgical Society. Two of these are of especial note, the "Vis Medicatrix Naturæ," this was the Presidential Inaugural Address of the Annual Meeting of the Bath and Bristol Branch of the British Medical Association, 1909, and the other was entitled "The Medical Life," an address given at the opening of the 29th session of the Bristol Medico-Chirurgical Society. In the meetings of these Societies he took much interest, was rarely absent from them, and in due course became President of both. These addresses are teeming with humour, and were much enjoyed by those present, in fact it was always expected that if Munro Smith was asked to give any sort of address or after dinner speech that it would be quite the event of the occasion; it is no exaggeration to say that he was truly a "born wit." He was not a voluminous or tedious writer, but the list of papers appended shows that his pen did much useful work and was frequently in demand.

The great work which has occupied the last year or two of his life is one which has yet to be published, but is of considerable note. It is no less than *A History of the Bristol Royal Infirmary*, a handsome volume of some 500 pages, which the author was fortunately able to finish before his health failed him, and which will be a valuable record of the foundation and history of an Institution which is one of the oldest provincial hospitals of this country. It is founded mainly on a bulky row of volumes, fourteen in number, labelled "Biographical Memoirs," which were compiled by the late Richard Smith, who was Surgeon to the Infirmary from 1796 to 1843. Munro Smith states in the Introduction that "it appeared to me that if these memorials of bygone times could be brought from their retreat into daylight, and be put into some kind of sequence and order, they could not fail to be of interest to many, not only as a history of a great charity, but as a means of looking with the eyes of a keen observer into the vivid life of an eventful epoch." The Committee, recognising his devotion to the Infirmary and his unique knowledge of the Institution for so many years, asked him to undertake this duty, which he consented to do. The volume was, however, delayed by the war, but the author had done his work, and this History will be a lasting memorial of his labours.

Of late much of his time had been taken up with work for the army. As one of the officers of the 2nd Southern Hospital, he was called up for duty at the commencement of the war, and the heavy addition to his work entailed by the office of Lieut.-Colonel, R.A.M.C.T., necessitated daily attendance for many hours at the Southmead War Hospital, where he devoted himself heartily to the patriotic work of the treatment of the large number of wounded soldiers which passed to his care. It must have been an arduous task to carry out such a charge becoming a commissioned officer, with various rights, privileges and duties, and yet continue to carry on the usual hospital work and that of private practice as heretofore, but Munro Smith did all this with credit to himself and to the satisfaction of his senior officers. His old friend and life-long colleague, Lieut.-Colonel Paul Bush, in command of the Second Southern Hospital, writes that he was one of the first to join the Territorial Force when inaugurated, and with the onset of war he was from the first most active in the work. "It was most noticeable how readily he adapted himself to the conditions and demands of military service, and was always ready to fall in with duties which are in many respects so different from those we are accustomed to in civil life. He was a most genial and helpful colleague, of whom it is difficult to speak too highly, and I personally mourn his loss as one of my oldest friends."

We often hear of the word "fame" in connection with medicine; but Munro Smith himself remarked in his address to the Medico-Chirurgical Society, "The fame acquired in our profession does not as a rule last very long." It is often said that, especially in army work, "this is now the day of young men," the old doctor is not so much in request. As was said by Rockwell (*Med. Rec.*, 1916, ii. 1109), "Great physicians seem to loom up larger in the past than in the present," but nevertheless our lost friend, George Munro Smith, will be held in grateful memory, which will be kept alive for many years in many grateful hearts through a large circle of friends, patients, and colleagues, by whom he will be greatly missed, and we may all hope, as he says, to

"join the choir invisible
Of those immortal dead, who live again
In lives made better by their presence."

BIBLIOGRAPHY.

"Notes on some Bristol Medical Societies," *Bris. Med.-Chir. J.*, 1914, xxxii. 268–286.

"Cholera Epidemics in Bristol in the Nineteenth Century," *Brit. M. J.*, 1915, ii. 60.

"Herpes and Brachial Neuritis," *Med. Press and Cir.*, 1912, N.S., xciv. 537.

"Renovation of Terminal Phalanx of Thumb," *Brit. M. J.*, 1910, ii. 1853.

"Vis Medicatrix Naturæ," *Bris. Med.-Chir. J.*, 1909, xxvii. 321-326.

"Case of Rupture of Middle Meningeal Artery, Operation, Recovery," *Bris. Med.-Chir. J.*, 1908, xxvi. 216–218.

"A Case of Mesenteric Thrombosis," *Bris. Med.-Chir. J.*, 1906, xxiv. 216–218.

"The Medical Life," *Bris. Med.-Chir. J.*, 1902, xx. 289–304.

"A Case of Perforated Duodenal Ulcer," *Bris. Med.-Chir. J.*, 1903, xxi. 218–220.

"The Spontaneous Disappearance of a Sarcomatous Tumour," *Bris. Med.-Chir. J.*, 1900. xviii. 30–33.

"A Case of Noma of the Ear," *Brit. M. J.*, 1898, ii, 714.

"A Case of Dislocation of Peronei Tendons Treated by Operation," *Brit. M. J.*, 1897, i. 1216.

"Pneumococci in the Urine," *Bris. Med.-Chir. J.*, 1895, xiii. 115.

"Displacement of the Ulnar Nerve," *Brit. M. J.*, 1893, vol. i. p. 228.

"Observations of the Normal Diet," *Bris. Med. Chir. J.*, 1889, vii. 73–77.

"On the Varieties of Hepatic Cirrhosis," *Brit. M. J.*, 1888, i. 1381.

"The Cardiograph in Medicine," *Bris. Med. Chir. J.*, 1883, i. 71–81.

## Figures and Tables

**Figure f1:**